# Consumer preference analysis on the attributes of samgyeopsal Korean cuisine and its market segmentation: Integrating conjoint analysis and K-means clustering

**DOI:** 10.1371/journal.pone.0281948

**Published:** 2023-02-16

**Authors:** Ardvin Kester S. Ong, Yogi Tri Prasetyo, Armand Joseph D. Esteller, Jarod E. Bruno, Kathryn Cheska O. Lagorza, Lance Edward T. Oli, Thanatorn Chuenyindee, Kriengkrai Thana, Satria Fadil Persada, Reny Nadlifatin

**Affiliations:** 1 School of Industrial Engineering and Engineering Management, Mapúa University, Intramuros, Manila, Philippines; 2 International Bachelor Program in Engineering, Yuan Ze University, Chung-Li, Taiwan; 3 Department of Industrial Engineering and Management, Yuan Ze University, Chung-Li, Taiwan; 4 Young Innovators Research Center, Mapúa University, Intramuros, Manila, Philippines; 5 Department of Industrial Engineering and Aviation Management, Navaminda Kasatriyadhiraj Royal Air Force Academy, Bangkok, Thailand; 6 Entrepreneurship Department, BINUS Business School Undergraduate Program, Bina Nusantara University, Malang, Indonesia; 7 Department of Information Systems, Institut Teknologi Sepuluh Nopember, Kampus ITS Sukolilo, Surabaya, Indonesia; King Faisal University, SAUDI ARABIA

## Abstract

Samgyeopsal is a popular Korean grilled dish with increasing recognition in the Philippines as a result of the Hallyu. The aim of this study was to analyze the preferability of Samgyeopsal attributes which includes the main entree, cheese inclusion, cooking style, price, brand, and drinks using Conjoint Analysis and market segmentation using k-means clustering. A total of 1018 responses were collected online through social media platforms by utilizing a convenience sampling approach. The results showed that the main entrée (46.314%) was found to be the most important attribute, followed by cheese (33.087%), price (9.361%), drinks (6.603%), and style (3.349%). In addition, k-means clustering identified 3 different market segments: high-value, core, and low-value consumers. Furthermore, this study formulated a marketing strategy that focused on enhancing the choice of meat, cheese, and price based on these 3 market segments. This study has significant implications for enhancing Samgyeopsal chain businesses and helping entrepreneurs with consumer preference on Samgyeopsal attributes. Finally, conjoint analysis with k-means clustering can be utilized and extended for evaluating food preferences worldwide.

## 1. Introduction

The Korean influence has been a trending topic worldwide. It was started around the late 1990s or widely known as the “Hallyu Wave” when Korean television dramas gained their first popularity [1]. This popularity led to the acknowledgment of Korean music, entertainment, and even Korean cuisines [2–5]. There are numerous Korean cuisines that are recognized worldwide as a result of the Korean influence and one of the most distinguishable dishes is Samgyoupsal [6].

Samgyeopsal is a popular type of grilled dish from Korean cuisine and it is mentioned as one of the most favored Korean cuisines. Its prepared ingredients are served while the customer cooks and assembles the dish by personal preference [6]. This cooking-eating style is able to sustain social interaction with companions and fellow customers are said to be significant factors in the popularization of Samgyeopsal among foreigners [7]. The impact of Samgyeopsal in different countries and its recognition has led to the opening of numerous Samgyeopsal restaurants in foreign countries like the U.S. [8], Australia [9], and UAE [10]. Additionally, one of the leading countries that have seen a significant increase in Samgyeopsal restaurants is the Philippines [11, 12].

The Philippines saw an 80 percent increase in Korean restaurants during the last decade, with Samgyeopsal at its center [13]. The trend of Samgyeopsal in the Philippines was partly influenced by its aspect of being a family-style dish, including the unlimited package deals, with reasonable prices, and the overall experience that the Samgyeopsal brands in the Philippines offer [6, 14]. Popular Samgyeopsal restaurants in the country include Sariwon Korean Restaurant, Soban K-Town Grill, Leann’s Tea House, Geonbae Modern Korean Bar & Grill, Samgyupsalamat, Romantic Baboy, and Seoul Train Korean BBQ [15].

A recent report done by [16] showed that the food trend in the Philippines went from the obsession of people for chocolate boutiques in 2010, a craze for milk tea in 2011 [17], Japanese influence of Ramen in 2012, Cronuts in 2013, everything related to craft food in 2014, healthy and whole food in 2015, salted egg and purple yam (*ube*) flavored food in 2016, Samgyeopsal in 2017 continuing in the present, and milk tea again in 2018 to present [17]. However, the COVID-19 pandemic has affected indoor dining which affected greatly the craze of Samgyeopsal and their offering of unlimited food service. To which, people shifted their craze to Baked Sushi due to its convenience and food delivery option [18]. Apparently, the Samgyeopsal offering ala carte was not a popular option among people. As indicated by [19], the Korean influence heightened the younger generation. Without the feel, ambiance, and unlimited food, it was seen that consumers had reduced consumption of the crazed Samgyeopsal in the Philippines. With the current lightening of the COVID-19 pandemic protocols, indoor dining opened doors to the consumers of Samgyeopsal. Businesses in the Philippines however have made ways to cater to customer needs; but have been challenged with what strategies are needed to promote their brand [20].

The popular restaurants diversified the different attributes they offer to highlight their differences from other restaurants. Some of these attributes are the main meat, the addition of cheese, cooking style, price range, brands, and even drinks. These attributes refer to the combination of options a customer may preferably choose when ordering in a Samgyeopsal restaurant. With numerous varieties of attributes being available, proper analysis must be conducted to deduce consumer preference. The knowledge in gaining consumer preference would lead to the creation of market segmentation and strategies from customer preferences. In addition, the change in consumer behavior when it comes to indoor dining has challenged the food industry [21, 22]. With the analysis of consumer preference, business development may be applied to enhance consumption in the food sector [23–25]. In this case, it would enable a Samgyeopsal restaurant to have a competitive advantage in the increase of competitors. Customer preference for Samgyeopsal can be analyzed by measuring the importance level of each attribute such as the conjoint analysis approach [17].

Conjoint Analysis is a popular strategy used by marketers to determine how consumers choose between rival items and suppliers [26–32]. It is frequently praised as a highly effective method for gathering precise information and conducting market research. It is also a method of determining how much customers value a product’s quality [33]. Conjoint analysis is usually done in the form of a customized survey in which consumers are asked to rate a particular aspect [34]. Several studies have utilized conjoint analysis in market segmentation and consumer preference. In line with the purpose of this research, conjoint analysis can be used in assessing consumers’ preferences regarding the specific attributes of a food product [17].

Many studies have used the conjoint analysis approach in the field of food-related marketing. For instance, [17] used conjoint analysis to understand which attribute of milk tea was most preferred by Filipino consumers. The results showed that pearl size had the highest average importance score followed by sugar level, ice, type of milk tea, price, the inclusion of cream cheese, and brand in order. A similar conjoint analysis approach was utilized by [35] in estimating the consumers’ willingness to pay for non-organic rice. The study accounted for a price, reduced health risk, farm environment quality, eating quality, certification, and fair trade, where attribute significance depended on the respondent’s demographic profile [35]. [36] also investigated the relevance of attributes for Uraro cookies. The attributes were flavor, texture, size, label, and packaging, ranked by highest to lowest significance. Moreover, [37] investigated the relationship between depression with food preference among elderly inmates. It concluded that depression has a significant positive effect on food preference, notably taste, texture, color variation, and dietary diversity. With different works of literature reviewed, there was no conjoint analysis research about the Samgyeopsal trend in the Philippines. In addition to conjoint analysis, market segmentation will also help to provide clustering of consumers based on the analysis data [38].

Market segmentation involves the process of segmenting customers into appropriate groups that fit their interests [39, 40]. [40] stated that through market segmentation, the researchers will be able to pinpoint the target customers, for instance, the Samgyeopsal restaurants. Commonly, four categories of market segmentation are considered: demographic, geographic, income, and behavioral segments. Three criteria, namely the common needs, uniqueness, and similarity of responses, are generally used in identifying different market segments [39]. For market segmentation, a common tool being utilized is a machine learning algorithm known as K-Means clustering.

K-Means utilizes a machine learning algorithm, commonly using Python as the programming language due to Python’s efficiency in machine and deep learning projects. The main goal of using K-Means is to associate similar data points and understand the underlying patterns presented. A study conducted by [41] utilized the K-Means algorithm in grouping the respondents based on stimuli ranking with Iranian food as an attribute. The study used conjoint analysis in identifying the similarities of each of the two clusters and segmented the respondents in order to create a new Iran food menu based on customer preference [41]. The results showed a significant difference in cluster utility scores of beans and rice, ghorme sabzi, banana, and cola; while roast chicken, kebab, caramel, and yogurt were favored in both segments [41]. Relatively, [42] aimed to identify combinations of attributes that affected consumers’ buying behavior of banana chips through conjoint analysis and segmentation of respondents. Out of the six attributes such as the amount of content, form of cuts, nutrition information, packaging material, how to bring the packaging, and flavors, only nutrition information was excluded due to its low percentage in the conjoint analysis stage. The study concluded with three clusters which were based on the respondents’ importance level of data, prioritizing variants, packaging materials, and amount of content [42].

With that, the aim of this study was to determine the preference for Samgyeopsal attributes among Filipino consumers utilizing a conjoint analysis approach. Specifically, the attributes such as the main entrée, cheese, style of Samgyup, price, brand, and drinks were considered. In addition, this study aimed to utilize K-Means clustering for market segmentation through demographics and the attributes of Samgyoupsal. The results of this study will impact the future decisions of Samgyeopsal restaurant owners and entrepreneurs regarding restaurant management since this study focused on the consumers’ preferences on the corresponding attributes. In addition, strategies may be created from the market segmentation and importance level seen from the results of this study. The strategy created may eventually lead to an increase in dining customers due to satisfaction and eventually lead to an increase in sales and popularity. The attributes considered in this study may also be utilized by other Samgyeopsal present in other countries and other restaurants for their market segmentation.

## 2. Conjoint design

This study was approved by Mapua University Research Ethics Committee. The respondents were asked to fill out a consent form which indicates that the responses and information gathered will solely be used for academic and research purposes, following the Data Privacy Act or Republic Act No. 10173 in the Philippines. Prior to the data collection, informed consent was obtained from all participants through signing the form.

Table 1 presents the attributes and levels considered in this study. There are a total of 6 attributes namely: the main entrée that considered beef, pork, chicken, and seafood as levels, the availability of cheese, the style of cooking such as pre-cooked, Hot Pot, and Grilled, the prices, brands, and the drinks.

**Table 1:** Attributes and levels.

Attributes	Levels
Meat	Pork, Beef, Chicken, Seafood
Cheese	with cheese, without cheese
Style	Hot pot, Grilled, Pre-cooked
Price	499 PhP (9.97 USD), 599 PhP (11.97 USD), 699 PhP (13.96 USD)
Brand	Romantic Baboy, Samgyupsalamat, Sariwon Korean Barbecue, Soban K-Town Grill
Drinks	Soju/Beer, Soft drinks, Juice

The first attribute of Samgyeopsal considered in this study was the meat. The meat is the main entrée of Samgyeopsal where the experience revolves around [6]. Four levels of meat were considered for this attribute such as pork, beef, chicken, and seafood. The meat of choice is an essential factor that may influence consumers’ inclination to eat Samgyeopsal. Samgyeopsal offers a variety of meat, with pork being a popular choice among consumers [43]. The article by [44] stated that consumers preferred pork belly, beef belly, and any boneless part of the chicken when eating Samgyeopsal. In addition, [45] and the Bellevue Korean Restaurant [46] indicated the meat of seafood preferred the ones that could be grilled and included in Hot Pot dishes. These meats are usually being offered in Samgyeopsal restaurants in the country, Philippines.

The second attribute of Samgyeopsal was the addition of cheese. Cheese can be both a stand-alone dish and an ingredient in cooking. Cheese could be used in making sauces, toppings, or as an accompaniment to soups. It has an essential role in several international cuisines and has the potential to completely change the taste of the dish due to its aroma and flavor [47]. The choice of cheese varies depending on the Samgyeopsal restaurant. Examples of cheeses used in Filipino Samgyeopsal restaurants are cheddar in Gen Korean BBQ and mozzarella in Romantic Baboy [47] and Premier The Samgyupsal [48]. In addition, the use of cheese in Samgyeopsal restaurants is not at all unique in the Philippines. Cheese is also used in countries such as the United States, where "MEET Korean BBQ" and "Kang Ho Dong Baekjeong" serve corn cheese alongside the main dishes. Even in Thailand, "Uki Korean BBQ" serves cheese dipping with Samgyeopsal, similar to the Philippines.

The third attribute considered in this study was style. Style refers to the way the main entrée is being prepared. The style level consists of Grilled, Hot Pot, and Pre-cooked. Grilled Samgyeopsal is the most popular style of how Samgyeopsal is eaten [49]. [49] explained that normally, unseasoned and plain slices of pork belly are grilled on a special pan or barbecue grill meant to take away fat. This is then served with a variety of condiments before being wrapped in lettuce for consumption. The other level considers the Hot Pot which is made up of thinly sliced meat and small vegetables cooked in a steaming hot broth and served in the center of a grilling pan [50]. The last style level is Pre-cooked Samgyeopsal, wherein the Samgyeopsal will be cooked by the staff before it is served to the customer.

The fourth attribute was the price. Price plays a vital role in affecting the buying decision of customers [17, 51]. The affordable price of an unlimited meat package is one of the reasons why Samgyeopsal became popular among Filipinos [14]. Specifically, three levels were used with a fixed price point: 499 PhP, 599 PhP, and 699 PhP following the top restaurants in the Philippines [15]. These package costings primarily depend on the quality of meat being served and the number of meat choices presented on their menu. Following the different restaurants offering Samgyeopsal, [52] showed that most of the restaurants offered these price ranges anywhere in the country. Averaging to a low of at least 499 PhP for good quality meat, the highest would be 699 until more than 1000 PhP. Therefore, only the top popular restaurant prices were considered in this study following [15].

**Fig 1:**
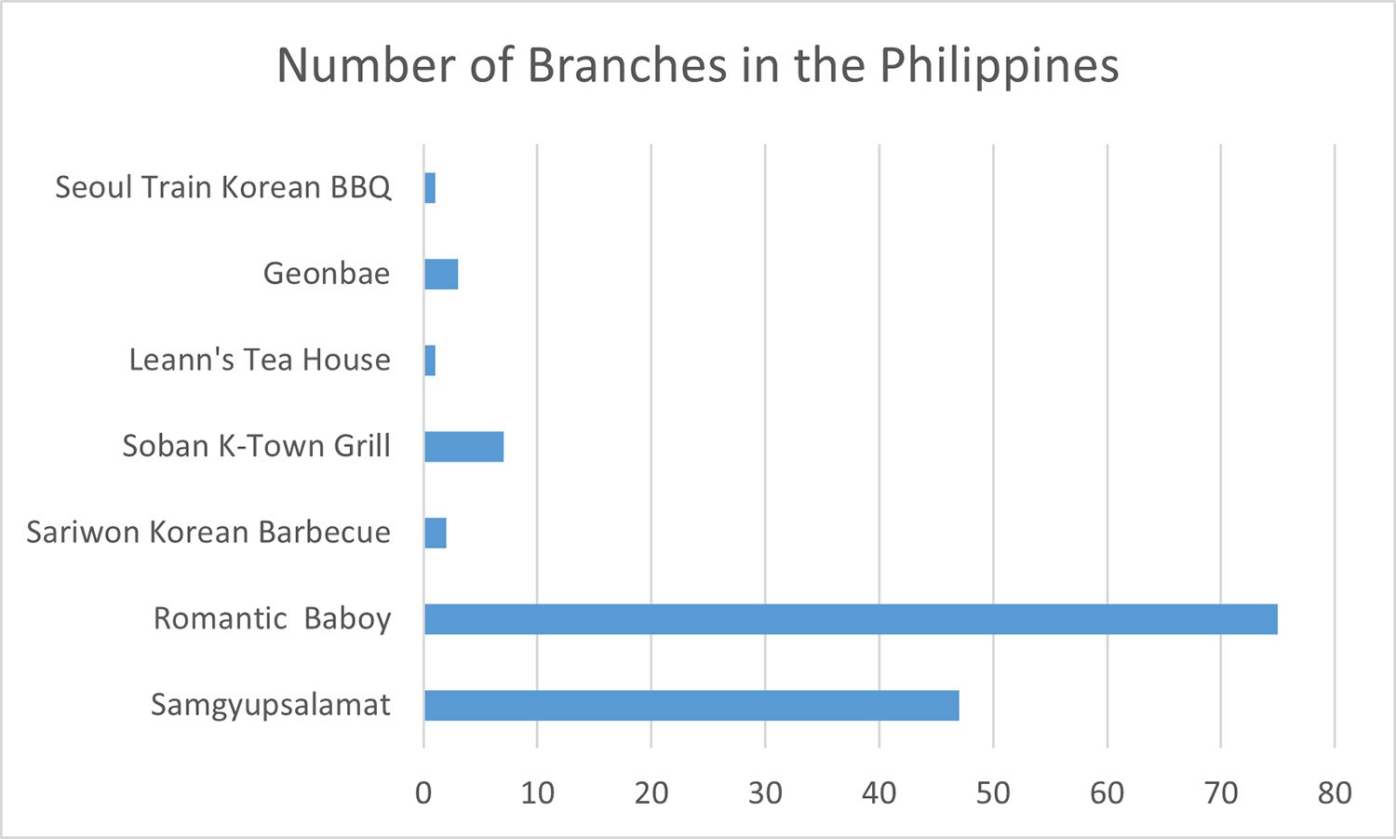
Number of Samgyeopsal branches of seven restaurants in the country [15].

The fifth attribute was the brand. The brand contains all the features of a business’s image and directly correlates with the product. The analysis done by [53] considered the effects of brand reputation in the catering industry. They considered customer satisfaction, purchasing intention, and brand loyalty which showed a positive significant effect on brand importance. Four brand levels were selected by popularity such as Sariwon Korea, Soban K-Town Grill, Samgyupsalamat, and Romantic Baboy [15]. These brands were listed among the top seven Samgyeopsal restaurants in Metro Manila as seen in Fig 1. From Fig 1, Sariwon Korea placed first, Soban K-Town Grill in second, Samgyupsalamat in fifth, and Romantic Baboy in sixth [15]. Compared to the number of branches of Romantic Baboy and Samgyupsalamat, the other five were listed as restaurants with the best Samgyusal due to the high-quality meat and overall food experience [15]. However, Romantic Baboy and Samgyupsalamat dominated the market with a lot of branches throughout the country compared to other brands.

The last attribute of Samgyeopsal was the drinks. Meals are always paired with drinks in order to assist in the consumption of food. Drinks help in the movement of food during digestion and provide optimal hydration [17]. Three levels were chosen for drinks: Soju/beer, soft drinks, and juice. Their commonality of being served in Samgyeopsal restaurants is the reason these drinks were considered for the level. Soju is the best drink to pair with Samgyeopsal, while soft drinks help in aiding the overall digestion process [54], as well as juices.

With the different attributes and levels considered, presented in Table 2 are the stimuli for the conjoint analysis with orthogonal design. There was a total of 27 stimuli generated using SPSS 25 with 2 holdouts following the study of [17, 55]. The addition of holdouts would entail consistency and reliability among the response gathered for this study [34, 55].

**Table 2:** Stimulus.

Combination	Meat	Cheese Inclusion	Style	Price	Brand	Drinks
1	Seafood	Without Cheese	Hotpot	599 PhP (11.97 USD)	Romantic Baboy	Juice/Iced tea
2	Beef	With Cheese	Hotpot	699 PhP (13.96 USD)	Soban K-Town Grill	Soft Drinks
3	Chicken	With Cheese	Pre-Cooked	499 PhP (9.97 USD)	Romantic Baboy	Soft Drinks
4	Chicken	Without Cheese	Hotpot	499 PhP (9.97 USD)	Soban K-Town Grill	Soju/Beer
5	Pork	With Cheese	Hotpot	499 PhP (9.97 USD)	Romantic Baboy	Soju/Beer
6	Seafood	With Cheese	Pre-Cooked	699 PhP (13.96 USD)	Sariwon Korean Barbecue	Soju/Beer
7	Seafood	With Cheese	Grilled	499 PhP (9.97 USD)	Romantic Baboy	Soju/Beer
8	Chicken	With Cheese	Grilled	599 PhP (11.97 USD)	Sariwon Korean Barbecue	Soft Drinks
9	Chicken	With Cheese	Hotpot	499 PhP (9.97 USD)	Sariwon Korean Barbecue	Juice/Iced tea
10	Pork	Without Cheese	Pre-Cooked	599 PhP (11.97 USD)	Soban K-Town Grill	Soju/Beer
11	Pork	With Cheese	Grilled	499 PhP (9.97 USD)	Soban K-Town Grill	Juice/Iced tea
12	Seafood	With Cheese	Grilled	599 PhP (11.97 USD)	Soban K-Town Grill	Soft Drinks
13	Chicken	With Cheese	Hotpot	599 PhP (11.97 USD)	Samgyupsalamat	Soju/Beer
14	Chicken	Without Cheese	Grilled	699 PhP (13.96 USD)	Romantic Baboy	Juice/Iced tea
15	Pork	Without Cheese	Pre-Cooked	599 PhP (11.97 USD)	Romantic Baboy	Soft Drinks
16	Beef	With Cheese	Pre-Cooked	499 PhP (9.97 USD)	Samgyupsalamat	Juice/Iced tea
17	Beef	With Cheese	Hotpot	599 PhP (11.97 USD)	Romantic Baboy	Soft Drinks
18	Beef	Without Cheese	Grilled	499 PhP (9.97 USD)	Sariwon Korean Barbecue	Soju/Beer
19	Pork	With Cheese	Hotpot	699 PhP (13.96 USD)	Romantic Baboy	Soju/Beer
20	Seafood	Without Cheese	Hotpot	499 PhP (9.97 USD)	Samgyupsalamat	Soft Drinks
21	Pork	With Cheese	Grilled	499 PhP (9.97 USD)	Romantic Baboy	Soft Drinks
22	pork	With Cheese	Grilled	599 PhP (11.97 USD)	Romantic Baboy	Juice/Iced tea
23	pork	Without Cheese	Grilled	699 PhP (13.96 USD)	Samgyupsalamat	Soft Drinks
24	pork	Without Cheese	Hotpot	499 PhP (9.97 USD)	Sariwon Korean Barbecue	Soft Drinks
25	pork	With Cheese	Grilled	599 PhP (11.97 USD)	Samgyupsalamat	Soju/Beer
26	beef	Without Cheese	Grilled	599 PhP (11.97 USD)	Romantic Baboy	Soju/Beer
27	pork	With Cheese	Hotpot	599 PhP (11.97 USD)	Sariwon Korean Barbecue	Juice/Iced Tea

## 3. Results

### 3.1 Participants

A total of 1018 Samgyeopsal consumers voluntarily answered a self-administered survey online. The study utilized convenience sampling in gathering data through several social media platforms and was made available from September 2021 to November 2021. The respondents considered were Filipino consumers of Samgyeopsal, validated through responses. Data collection of conjoint analysis responses online and during the COVID-19 pandemic was deemed acceptable as evaluated by [55] and [56]. The statement, "I voluntarily agree to participate in the survey and have read the description above," was also prompted before continuing to the main section of the questionnaire.

### 3.2 Demographics

Presented in Table 3 are the descriptive statistics of the demographics. From the results, it was seen that 59.7% are female while 39.4% were male wherein the majority were from ages 15–24 years old (84.6%) followed by 25–38 years old (8.30%). Following the study of Blas and Erestain [19], those of the younger age group are the highly influenced group when it comes to Korean culture. In addition, Masigan [57] explored the age group in the trends of food industries in the Philippines. To their findings, those in the lower age bracket are the usual customers. In the case of Samgyeopsal in the Philippines, this kind of restaurant chain offers unlimited serving at an affordable price and thus also caters much to the students and working class of the respondents. In relation, the majority of the respondents had a monthly salary/allowance below 15,000 PhP (low-income group) with 74.4% followed by 15,000–30,000 PhP (13.2%) coming from the National Capital Region (NCR) with 63.4%, Region III (13.3%), and Region IV-A (17.2%). Lastly, the majority of the respondents eat Samgyeopsal at least once a week (66.3%), twice a week (22.1%), or thrice a week (7.10%).

**Table 3:** Descriptive statistics of demographics (N: 1018).

Characteristics	Category	n	%
**Gender**	Male	401	39.40
	Female	608	59.70
	Others	9	0.900
	15–24 years old	861	84.60
	25–38 years old	84	8.300
**Age**	39–44 years old	46	4.500
	45–54 years old	20	2.000
	More than 55 years old	7	0.700
	Less than 15,000 PHP	757	74.40
**Monthly Income/Allowance**	15,000–30,000 PHP	134	13.20
	30,001–45,000 PHP	81	8.000
	45,001–60,000 PHP	10	1.000
	More than 60,000 PHP	36	3.500
	CAR	6	0.600
	NCR	645	63.40
	Region I	12	1.200
	Region II	5	0.500
	Region III	135	13.30
	Region IV-A	175	17.20
	Region IV-B	5	0.500
**Location**	Region V	9	0.900
	Region VI	10	1.000
	Region VII	5	0.500
	Region VIII	2	0.200
	Region IX	2	0.200
	Region X	1	0.100
	Region XI	2	0.200
	Region XIII	4	0.400
	BARMM	0	0.000
**How many times do you eat Samgyeopsal in a week?**	1	675	66.30
	2	225	22.10
	3	72	7.100
	4	30	2.900
	5	6	0.600
	6	9	0.900
	7	1	0.100

### 3.3 Statistical analysis

The conjoint analysis with the orthogonal design utilized SPSS 25. A total of 27 stimuli were generated by the SPSS. The orthogonal design was utilized to ensure the reasonable number of stimuli that were evaluated by the participants. Table 4 presents the 27 stimuli evaluated by a 7-point Likert scale ranging from 1 as “strongly disagree” to 7 as “strongly agree”. From the results, it was seen that combination 11 with Pork as the main with cheese as dip, cooked through grilling priced at 499 PhP (9.97 USD) at Soban K-Town Grill with Juice or Iced Tea as the drink was the most preferred. On the other hand, combination 1 was the least preferred with seafood as the main entree without cheese, cooked through Hot Pot priced at 599 PhP (11.97 USD) at Romantic Baboy with Juice or Iced Tea as the drink the least preferred.

**Table 4:** Stimulus rank.

Combination	Meat	Cheese Inclusion	Style	Price	Brand	Drinks	Rank
1	Seafood	Without Cheese	Hotpot	599 PhP (11.97 USD)	Romantic Baboy	Juice/Iced tea	27
2	Beef	With Cheese	Hotpot	699 PhP (13.96 USD)	Soban K-Town Grill	Soft Drinks	11
3	Chicken	With Cheese	Pre-Cooked	499 PhP (9.97 USD)	Romantic Baboy	Soft Drinks	12
4	Chicken	Without Cheese	Hotpot	499 PhP (9.97 USD)	Soban K-Town Grill	Soju/Beer	24
5	Pork	With Cheese	Hotpot	499 PhP (9.97 USD)	Romantic Baboy	Soju/Beer	5
6	Seafood	With Cheese	Pre-Cooked	699 PhP (13.96 USD)	Sariwon Korean Barbecue	Soju/Beer	22
7	Seafood	With Cheese	Grilled	499 PhP (9.97 USD)	Romantic Baboy	Soju/Beer	16
8	Chicken	With Cheese	Grilled	599 PhP (11.97 USD)	Sariwon Korean Barbecue	Soft Drinks	13
9	Chicken	With Cheese	Hotpot	499 PhP (9.97 USD)	Sariwon Korean Barbecue	Juice/Iced tea	10
10	Pork	Without Cheese	Pre-Cooked	599 PhP (11.97 USD)	Soban K-Town Grill	Soju/Beer	20
11	Pork	With Cheese	Grilled	499 PhP (9.97 USD)	Soban K-Town Grill	Juice/Iced tea	1
12	Seafood	With Cheese	Grilled	599 PhP (11.97 USD)	Soban K-Town Grill	Soft Drinks	17
13	Chicken	With Cheese	Hotpot	599 PhP (11.97 USD)	Samgyupsalamat	Soju/Beer	14
14	Chicken	Without Cheese	Grilled	699 PhP (13.96 USD)	Romantic Baboy	Juice/Iced tea	25
15	Pork	Without Cheese	Pre-Cooked	599 PhP (11.97 USD)	Romantic Baboy	Soft Drinks	19
16	Beef	With Cheese	Pre-Cooked	499 PhP (9.97 USD)	Samgyupsalamat	Juice/Iced tea	6
17	Beef	With Cheese	Hotpot	599 PhP (11.97 USD)	Romantic Baboy	Soft Drinks	9
18	Beef	Without Cheese	Grilled	499 PhP (9.97 USD)	Sariwon Korean Barbecue	Soju/Beer	21
19	Pork	With Cheese	Hotpot	699 PhP (13.96 USD)	Romantic Baboy	Soju/Beer	8
20	Seafood	Without Cheese	Hotpot	499 PhP (9.97 USD)	Samgyupsalamat	Soft Drinks	26
21	Pork	With Cheese	Grilled	499 PhP (9.97 USD)	Romantic Baboy	Soft Drinks	2
22	pork	With Cheese	Grilled	599 PhP (11.97 USD)	Romantic Baboy	Juice/Iced tea	3
23	pork	Without Cheese	Grilled	699 PhP (13.96 USD)	Samgyupsalamat	Soft Drinks	18
24	pork	Without Cheese	Hotpot	499 PhP (9.97 USD)	Sariwon Korean Barbecue	Soft Drinks	15
25	pork	With Cheese	Grilled	599 PhP (11.97 USD)	Samgyupsalamat	Soju/Beer	7
26	beef	Without Cheese	Grilled	599 PhP (11.97 USD)	Romantic Baboy	Soju/Beer	23
27	pork	With Cheese	Hotpot	599 PhP (11.97 USD)	Sariwon Korean Barbecue	Juice/Iced Tea	4

The utility scores are presented in Table 5, followed by the average score of importance in Table 6. From the results, it could be seen that the Main Entrée and Cheese was the most preferred attribute for Samgyeopsal among Filipino consumers with values of 46.314% and 33.087%, respectively. The utilities from which showed that people highly preferred beef (0.425) and pork (0.216), and least preferred chicken (-0.168) and seafood (-0.473). Moreover, people preferred with cheese (0.321) rather than without cheese (-0.321). The other attributes were considered least important due to the difference in importance value. From which, Price (9.361%) was the highest, followed by Drinks (6.603%), Style (3.349), and Brand (1.287). For the prices, the utilities were not that far with 499 PhP or 9.97 USD as the most preferred (0.101), followed by 599 PhP or 11.97 USD (-0.020), then 699 PhP or 13.96 USD (-0.081). For the Drinks, Juice or Iced Tea (0.058) was the most preferred, followed by soft drinks (0.011), then Soju or Beer (-0.070) was the least preferred. The Style of cooking the main entrée differs from Grilled (0.037), followed by least preferred hot pot (-0.009) and Pre-cooked (-0.028). Lastly, the Brand had the least important score with SamgyupSalamat (0.014) as the most preferred, followed by Soban K-Town Grill (0.002), Romantic Baboy (-0.006), and then Sariwon Korean Barbecue (-0.010) as the least preferred.

**Table 5:** Utilities score.

Attribute	Level	Utility Estimate	Std. Error
	Pork	0.216	0.028
	Beef	0.425	0.035
Main Entrée	Chicken	-0.168	0.035
	Seafood	-0.473	0.035
Cheese	With	0.321	0.019
	Without	-0.321	0.019
	Hot Pot	-0.009	0.025
Style	Grilled	0.037	0.025
	Pre-cooked	-0.028	0.030
	499 PhP (9.97 USD)	0.101	0.025
Price	599 PhP (11.97 USD)	-0.020	0.025
	699 PhP (13.96 USD)	-0.081	0.030
	Romantic Baboy	-0.006	0.028
Brand	SamgyupSalamat	0.014	0.035
	Sariwon Koren Grill	-0.010	0.035
	Soban K-Town Barbecue	0.002	0.035
	Soju or Beer	-0.070	0.025
Drinks	Soft Drinks	0.011	0.025
	Juice or Iced Tea	0.058	0.030

**Table 6:** Importance score.

Attributes	Values
Main Entrée	46.314
Cheese	33.087
Price	9.361
Drink	6.603
Style	3.349
Brand	1.287

Table 7 presents the correlation of the conjoint analysis done. It could be seen that the correlation of Pearson’s R-value was high with a value of 0.991. This indicated that the attributes and levels were significant [34]. Moreover, the Kendall’s Tau value was 0.932. [34] indicated that values greater than 0.70 indicate significant results. Lastly, following the study of [55], 2 holdouts were added to validate the consistency of response. Based on the results, the Kendall’s Tau for holdout had a value of 1.000, interpreted as internal consistency among the responses.

**Table 7:** Correlation.

Analysis	Value	Significance
Pearson’s R	0.991	Less than 0.050
Kendall’s Tau	0.932	Less than 0.050
Kendall’s Tau for Holdouts	1.000	

### 3.4 K-Means clustering

**Fig 2:**
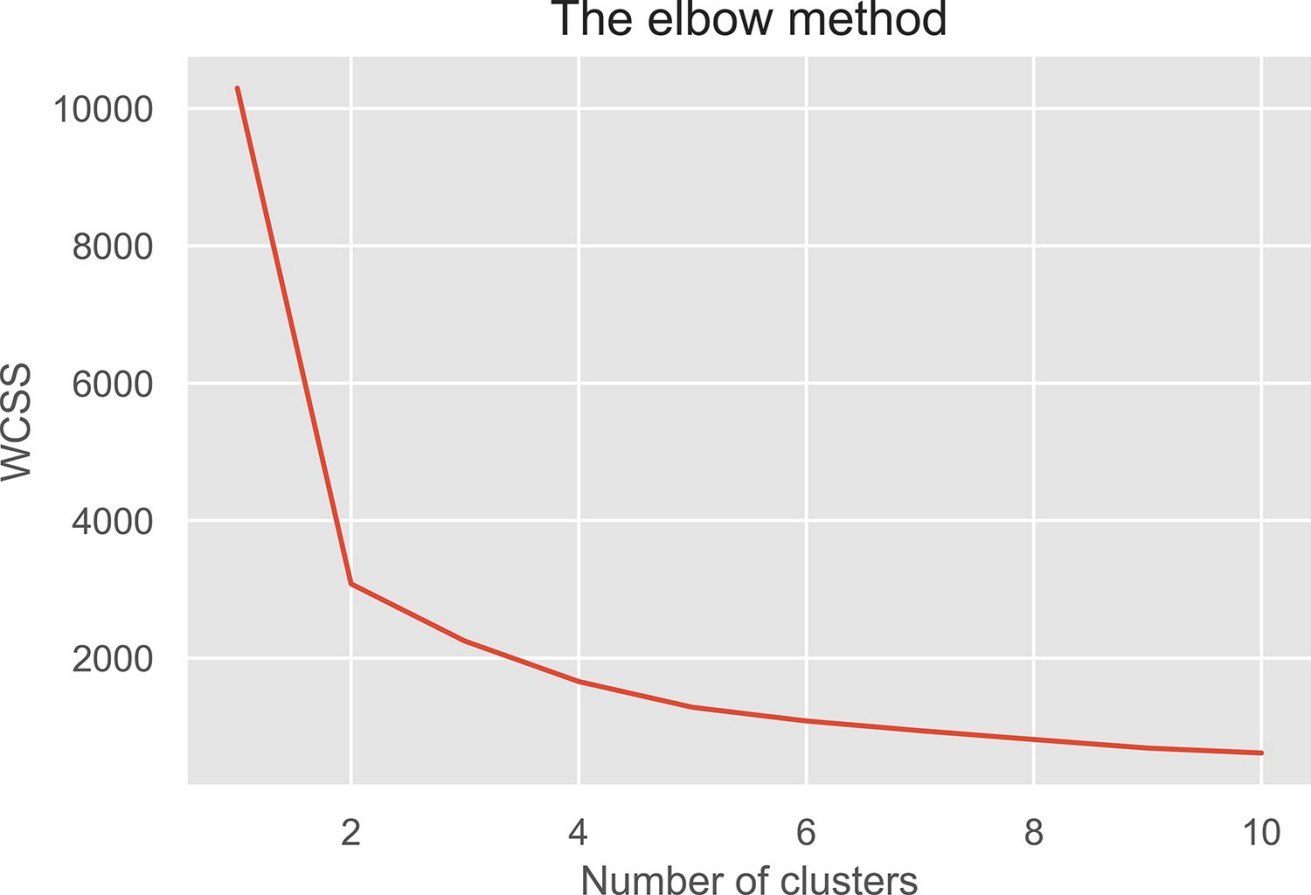
Elbow method plot.

Utilizing Python 3.8, K-Means clustering was conducted to determine the market segmentation of Filipino Samgyeopsal consumers. No missing data was found from the collected responses. The unsupervised data were clustered based on the demographics of gender, age, monthly allowance, or salary to the frequency of dining in Samgyeopsal. The data underwent a normalization process utilizing min_max scalar before processing the elbow method (Fig 2) was performed to determine the maximum cluster before the implementation of the K-Means algorithm.

**Fig 3:**
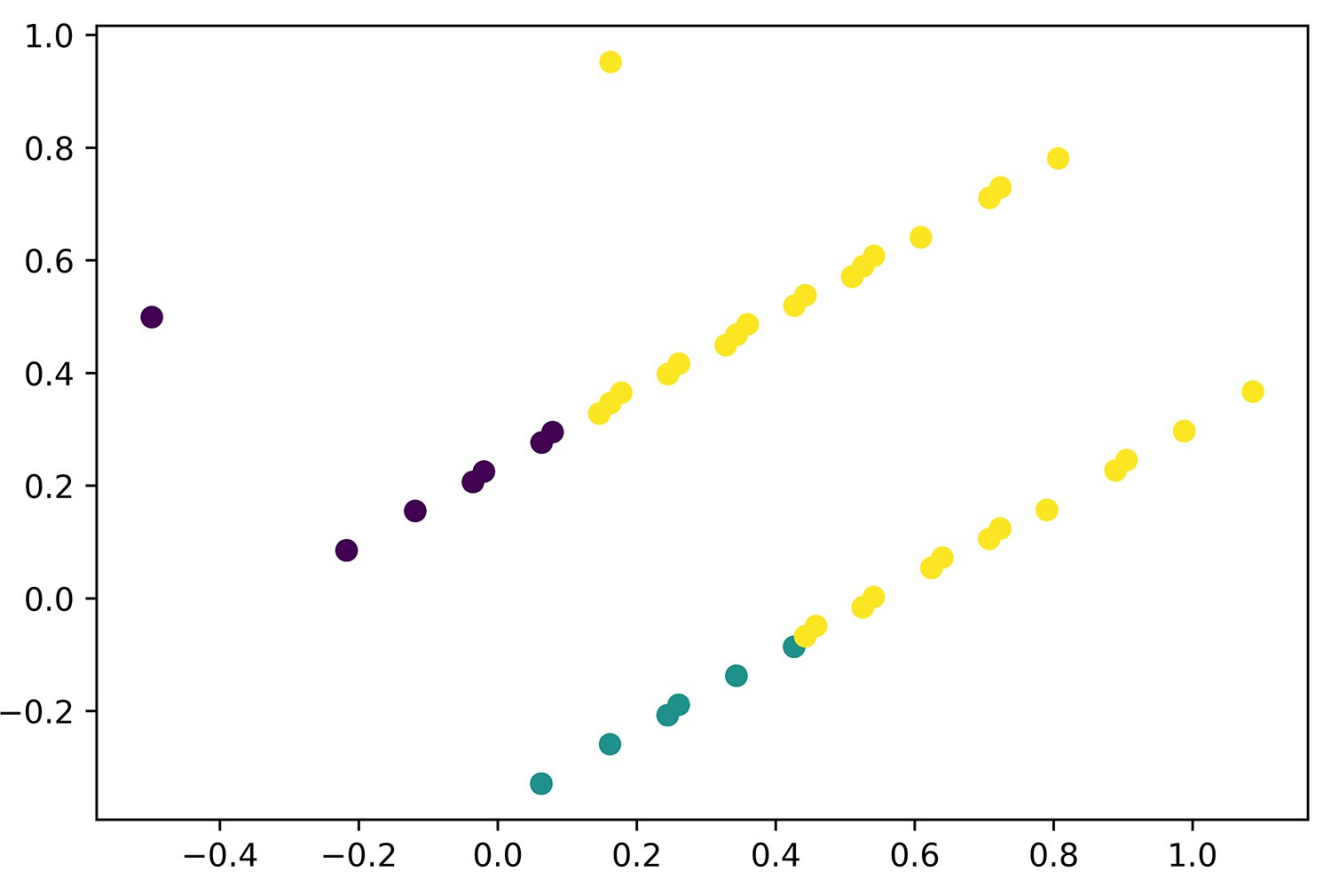
K-Means clustering.

**Fig 4:**
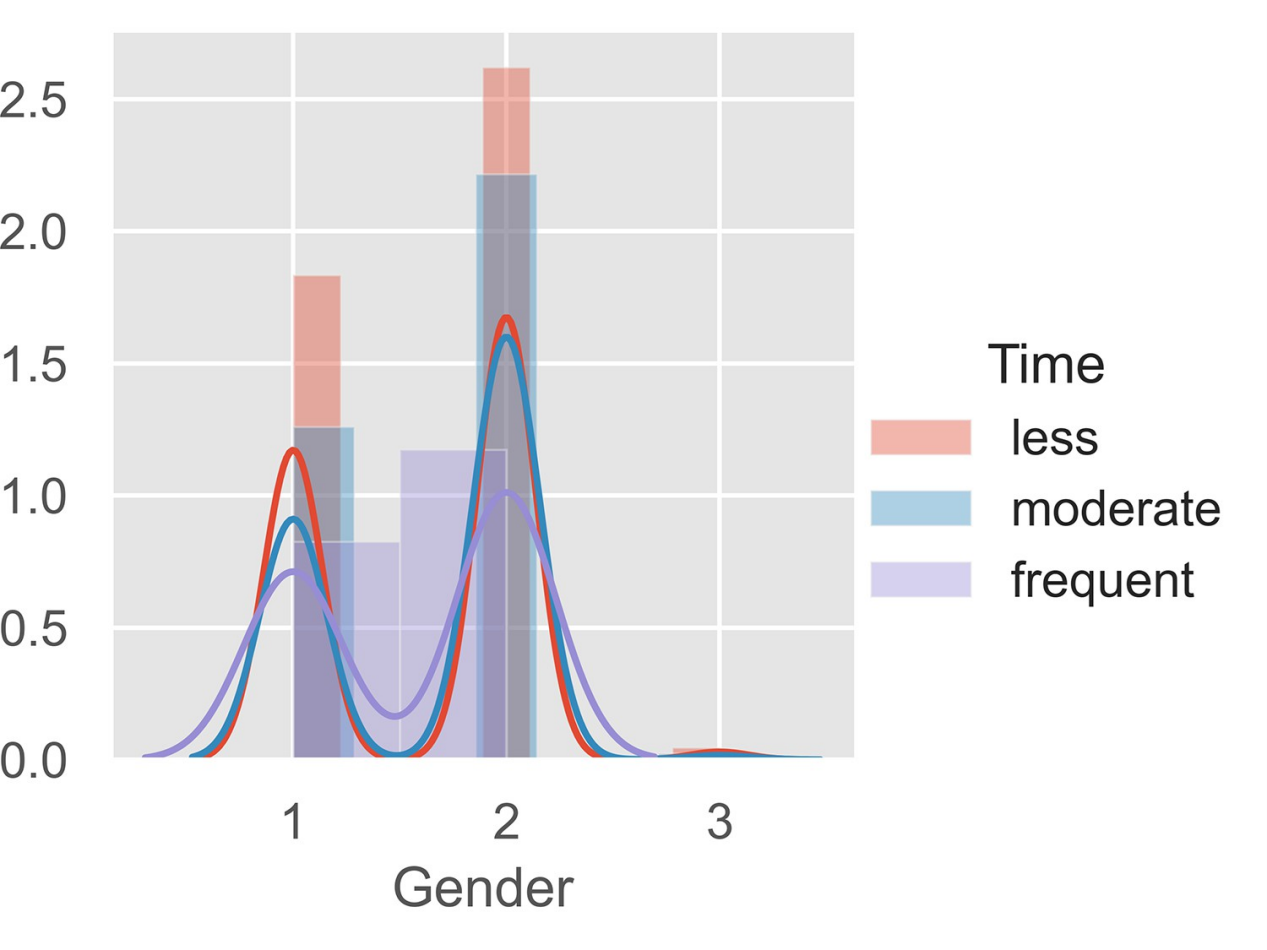
Cluster visualization through gender.

**Fig 5:**
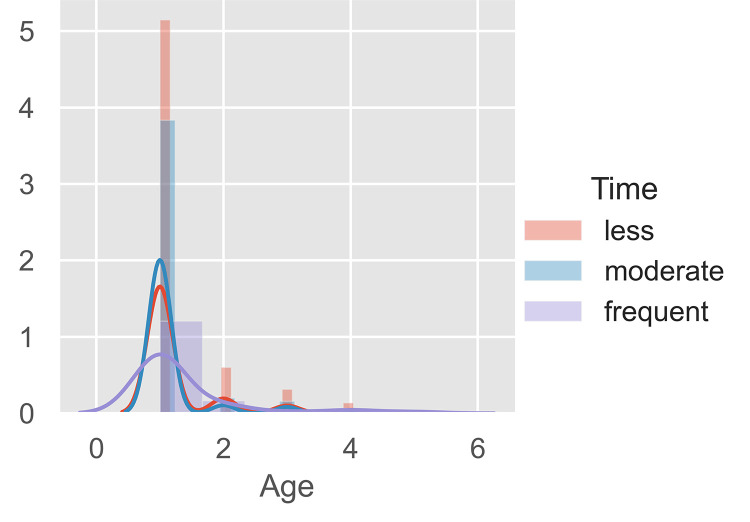
Cluster visualization through age.

**Fig 6:**
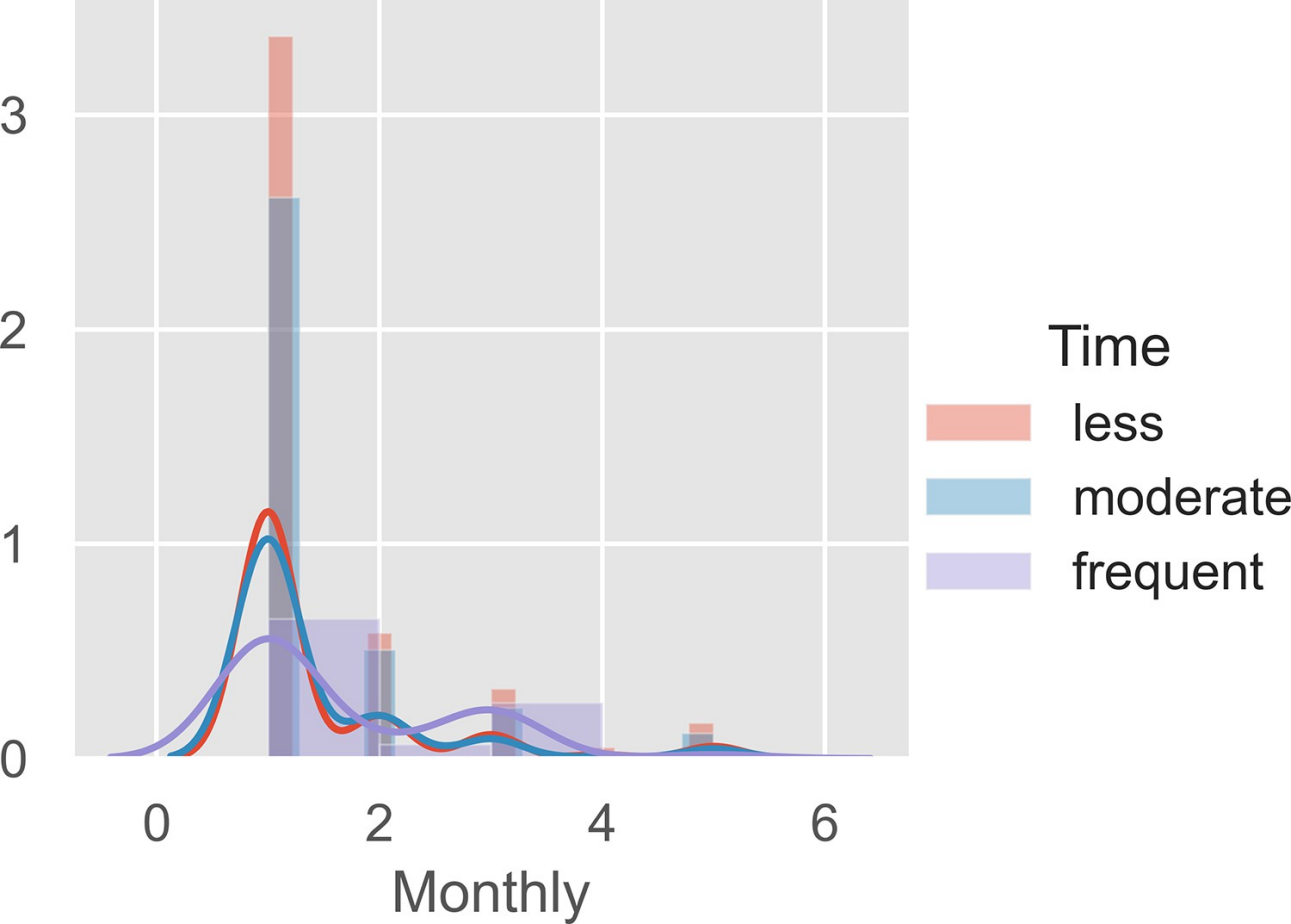
Cluster visualization through monthly allowance/salary.

From Figs 2 and 3 clusters could be made from the data collected. With 1000 iterations, the K-Means clustering of raw data was performed as seen in Fig 3. The cluster presents violet, yellow, and green which are the cluster of frequency versus monthly allowance or salary for dining in Samgyeopsal among Filipino consumers. To categorize the result, cluster visualization was performed as seen in Fig 4–6.

Presented in Fig 4 are the normalized dataset distribution of the gender of the respondents. It could be seen that most respondents who are female would less likely to consume Samgyeopsal compared to male. However, those who are frequent consumers are within both genders. Interestingly, the report of [57] deciphered trends in consumption for the food industry in the Philippines to be age-related rather than gender-related. Justifying the findings of this study, frequent consumers may either be of the male or female gender.

Presented in Fig 5 are the age range. Most of the respondents are seen in the lower age bracket with moderate consumption of Samgyeopsal to have frequent consumption as indicated by the blue and purple bars. In this case, the average age of the consumers of the food industry of the Philippines is said to be around 24.5 years old on the average [57]. In addition, [19] specifically discussed how the influenced group when it comes to the culture where Samgyeopsal originated are mostly of the younger generation which supports the findings of the study.

Fig 6 represents how those of lower income would tend to consume more. With the Samgyeopsal in the Philippines offering unlimited meat, side dishes, and rice among consumers, the benefit of eating a lot has been taken advantage of by the food clientele of the country. Having low income with the advantage of consuming a lot of food–and in relation, of a younger generation, would be highly beneficial for the consumers.

It could be deduced that gender had the least likely effect on Samgyeopsal consumption among Filipinos. However, the bulk of the respondents was of the lower-income bracket, below 15,000 PhP with 74.4% followed by 15,000–30,000 PhP (13.2%) and 84.6% of the respondents come from ages 15–24 years old. This presents how monthly allowance/salary and age affect the frequency or Samgyeopsal consumption in the Philippines. Younger generations are more likely to consume Samgyeopsal, but are bound to their budget. Appealing to the demographics, Samgyeopsal in the Philippines are required to ensure that they meet the budget of their consumers. In line with the conjoint analysis, it could be seen that prices are third among the importance score. Therefore, consumers are still inclined to Samgyeopsal dining.

## 4. Discussion

Presented in the discussion section of this paper is the evaluation of the conjoint analysis and K-Means clustering results. The discussion will expound on the collected data and additional information on an attribute’s importance score, the significance of each level, and market segmentation. The best combination would result in a 0.956 utility estimate for beef with cheese cooked through grilling with a price of 499 PhP (9.97 USD) at SamgyupSalamat with Juice or Iced Tea as drinks. The least preferred would result in -0.992 utility estimate for seafood without cheese, served by pre-cooked meat entrée priced at 699 PhP (13.96 USD) at Sariwon K-Town Grill with Soju or Beer as the drink.

### 4.1 Conjoint analysis

From the conjoint analysis result, the most preferred attribute of Samgyeopsal was meat (46.314%). Meat is generally associated with better wealth, taste, and luxury for humans [58]. Moreover, [6] stated that meat is usually considered the main characteristic of Samgyeopsal. Among the meat levels, the most preferred was beef (0.425), followed by pork (0.216), chicken (-0.168), and the least was seafood (-0.473). Beef is considered a symbol of wealth in Korean culture, making it a popularized choice in Korean cuisine and portrayed as a luxury in Korean popular culture [43]. With the Philippines being huge fans of Korean culture [11], Filipinos’ inheritance of this perception plays a factor in this study’s high preference for beef. Beef is also considered the most expensive type of meat among the considered levels from Korea and the Philippines [59, 60], and the least consumed meat by Filipinos out of the considered meats by a significant margin [61, 62]. Because beef is expensive and exquisite, this makes it the most desirable choice due to its higher perceived quality from consumers [63]. Thus, beef has been seen as the preferred meat among other choices.

The most desired meat following beef was pork. Pork became a highly preferred meat for Samgyeopsal because it is usually the most associated meat with the dish. Additionally, the preference for pork is high among Filipinos, considering it is the most popularly consumed meat in the Philippines [62]. It is also the most consumed meat in Korea aside from beef, naturally setting its place in Korean popular culture and influencing Filipinos’ meat preference for Samgyeopsal [11]. On the other hand, chicken was the less preferred meat for Samgyeopsal. Chicken in Korean cuisine is usually denoted as the meat being fried rather than being incorporated with the styles that Samgyeopsal offers, making it an unpopular choice for the dish [64]. This justifies how Korean culture respects and solidifies the origins and traditions of Korean cuisine and how Filipinos regard its portrayal in popular culture. Lastly, seafood was the least preferred meat for Samgyeopsal. Seafood generally has a negative connotation to many people due to its taste and smell. Additionally, it started gaining a bad reputation in recent years due to its source–becoming more polluted to spills and wastes, garnering negative criticism from professional health nutritionists which eventually affected customer preference for consumption.

The second highest attribute was the cheese (33.087%), wherein people preferred having cheese (0.321) over no cheese (-0.321) alongside their Samgyeopsal. One explanation points to the casein protein, responsible for releasing casomorphins that trigger strong addictive responses towards cheese. Salty is one of the key flavors in Filipino cuisine, alongside sweet [17]. This explains the high preference of Filipinos for cheese having high amounts of salt, especially cheap, processed cheeses, which are widely used in the country [17]. The combination of flavor and texture, along with its creaminess, salt, and tang, add up to the food experience just by adding cheese. Based on the results, it can be concluded that many Filipinos preferred the taste of Samgyeopsal with cheese due to its added flavor and texture.

The other attributes were considered the least important, showing great significance among the importance score. However, the Price (9.361%) would be considered the highest attribute among these, with its level being 499 PhP (9.97 USD), then 599 PhP (11.97 USD), and the least preferred was 699 PhP (13.96 USD). From the utility score, it was seen that each level is not that significantly different. This shows that the utility estimates of the different prices are relatively close to one another, therefore having a lesser effect on the Samgyeopsal preference. The demographics of a large percentage of the respondents of this study belong to the low-income group, which tends to lean toward lower product prices [65]. However, the price of a product directly relates to its quality, meaning that lower food prices also offer lower food quality [66].

Most Samgyeopsal restaurants in the Philippines have an affordable prices, considering the unlimited portions of the meal. Even if the Samgyeopsal is not of top quality, the restaurant could still entice many customers due to its low prices and serving quantity. In the Philippines, people’s preference when it comes to prices is affordability and budget-friendly [51]. The price levels observed only have a 100 PhP (1.96 USD) difference, thus would not be a significant distinction from each other. From observing the different offers of Samgyeopsal joints, a price increase can be deduced from the variety of choices offered. Variety in this context refers to the type of dish or how the meat was prepared and the number of side dishes available. A prime example would be Leann’s Tea House, with 399 PhP, 499 PhP, and 599 PhP. The differences are the types of dishes served per level and the addition of kimchi rice for the 599 PhP set. By observation, this principle also applies to other restaurants such as Samgyupsalamat, Geonbae, and K-Pub, adding variety to their menu and meat options such as more meat as their menu set–prices increase [15].

Drinks were also one of the low important scored attributes (6.603%). From the utility score, Juice or Iced Tea ranked first in preference (0.058), followed by soft drinks (0.011), and Soju or beer (-0.070). According to [67], the main consumers of tea and soft drinks in the Philippines are adolescents, which are also the highest number of respondents in this study. Also, fruit-flavored juice drinks were popular among preschool children [67]. This reasons why juice or iced tea and soft drinks have a higher significant value than soju or beer. According to the Tea Association of the U.S.A. [68], the most consumed beverage in the world next to water is tea. [69] described iced tea as an influential drink due to its sweetness and a "perfect summertime drink" due to it being cold and refreshing, which was also discussed in the study of [17]. It is common for Samgyeopsal restaurants to have a hot atmosphere because of the smoke and flames on every table, making iced tea a good complimentary drink for the set-up. Soft drinks are also a popular drink choice for Filipinos, where the country’s per capita yearly consumption of a leading brand is equivalent to almost 23 liters per person [51]. The popularity of this drink is also evident in the country’s consumption rate of soft drinks, which have the highest consumption compared to other non-alcoholic drinks [17]. On the other hand, soju is renowned for being the most famous alcoholic drink, with Jinro Soju having sold 86.3 million cases in 2019 [70]. Soju enhances both the flavors of Korean food and its spiciness. A survey also concluded that Samgyeopsal with soju is the best Korean food culture experience [71]. In Korean tradition, soju is meant to be paired with food and intended to be drunk with a group of people. However, based on the result, Filipinos would not tend to have soju with their Samgyeopsal. This result is evident in the statistics shown by [72], which presents the rankings of highly consumed drinks in the Philippines during 2018. Soda, juice, and tea are present in the top 7 selections, while soju falls under the category of “others” with a rating of 0.34%, the second-lowest rating next to “Prefer not to say” [62, 72].

Among the levels considered for the Style (3.349%), grilled was seen to be significant (0.037) while hotpots (-0.009) and pre-cooked (-0.028) were not considered as significant. This shows that grilled Samgyeopsal is the most preferred style of customer service. [73] stated that one of the main reasons why grilled is much preferred was because of the preparation and the addition of the savory and burnt taste of the meat. The most significant style attribute is grilled since this is how Samgyeopsal was introduced in the Philippines through Korean dramas. This explains why hotpots and pre-cooked Samgyeopsals were not that preferred by most Filipino consumers since cultural influence played a significant role. Samgyeopsal being popular in the Philippines has influenced businesses including Samgyupsalamat to shift an a la carte Korean barbecue restaurant to an unlimited meat restaurant because of the growing numbers of consumers.

The least important was the Brands (1.287%), where Samgyupsalamat (0.014) and Soban K-Town (0.002) were preferred more than Romantic Baboy (-0.006) and Sariwon Korean Barbecue (-0.010). The oldest among these brands is Samgyupsalamat, established in 2013, long before the Samgyeopsal trend in 2018 started [45]. Although currently known for its unlimited meat platters, Samgyupsalamat back in 2013 only offered individual orders of meat and ala carte meals [45]. The rise of popularity following the established Samgyupsalamat and Romantic Baboy franchise had left people with feelings of complacency towards the brand as it is most familiar to them. The paper of [74] related the buying frequency of consumers and brand loyalty, which supports the idea of the previous statement. That is why the popularity of Samgyupsalamat reverted to join the market with the unlimited eat-all-you-can in order to keep up with the market. In relation to [17], brands were also not that high among the different importance scores.

From the results, it was seen that people would consider highly the meat and the price when consuming Samgyeopsal in the Philippines. With that, further classification for market segmentation utilizing both attributes among the demographics of this study underwent K-Means clustering [38–40].

### 4.2 K-Means clustering

**Fig 7:**
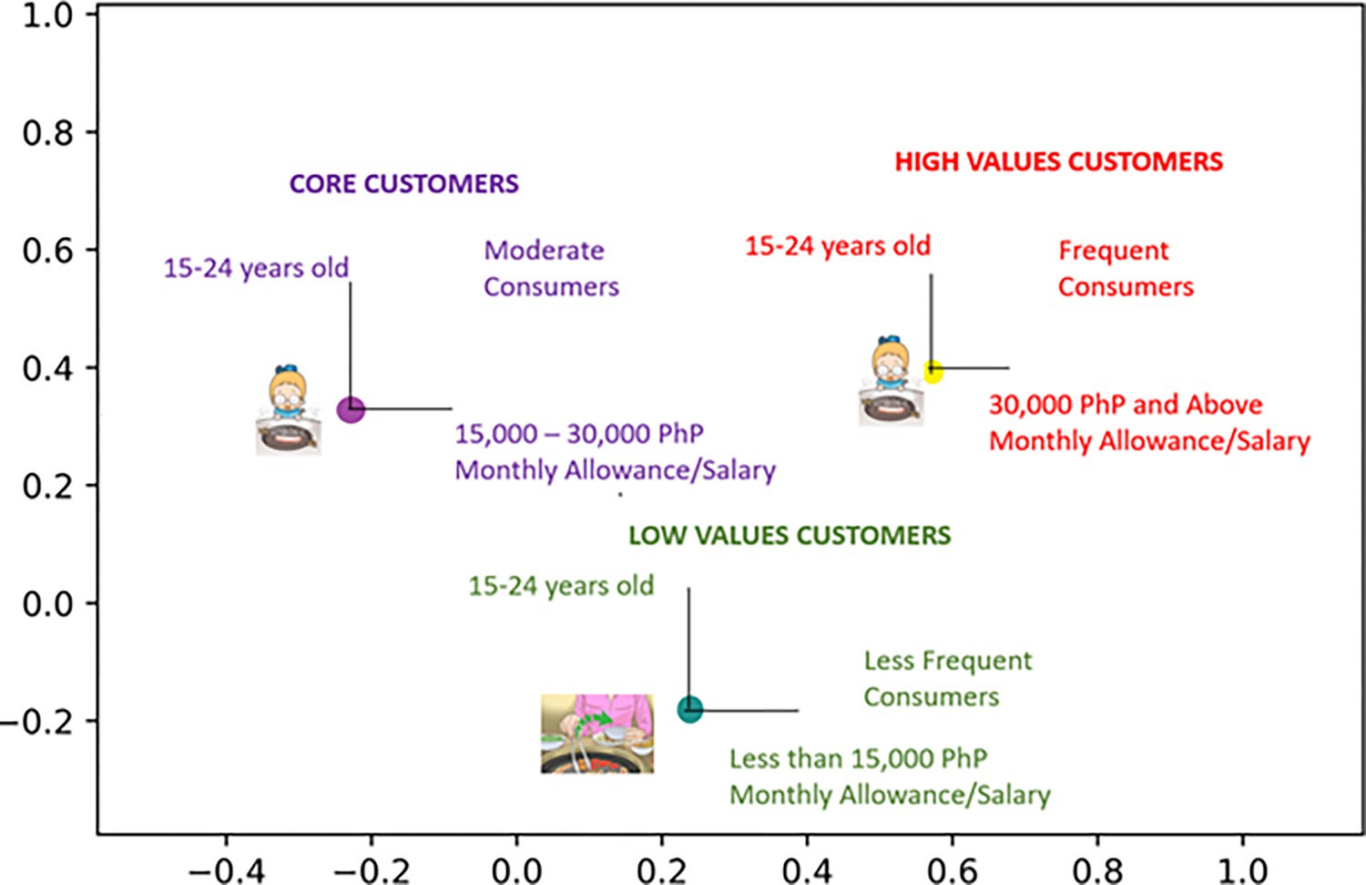
Graphical market segmentation.

Presented in Fig 7 is the graphical market segmentation from the demographics considered in this study. Specifically, age, monthly salary/allowance, and gender were utilized for the market segmentation [40]. The K-Means clustering using Euclidean distance metric from Python 3.8 to partition the consumers is seen in the figure. Moreover, the Silhouette Score was obtained with a value of 0.999 which indicates consistency among the clusters created.

From the market segmentation, the consumers are ages 15–24 years old. Wherein, the high-value consumers are the ones with 30,000 PhP and above monthly allowance/salary, the core customers have a monthly allowance/salary between 15,000–30,000 PhP, while the low-value customers have less than 15,000 PhP monthly allowance/salary.

#### 4.2.1 High Value Consumers

High values customers are categorized as buyers of ages 15–24 with more than 30,000 PhP monthly allowance/salary. Filipino habits or traditions of dining in Samgyeopsal restaurants in celebration of events, accomplishments, or birthdays is an example of high-value consumption. Celebrations, especially Filipino celebrations, are often incorporated with family gatherings and reunions. Food is always present in events, attested by Philippine fiestas, thus it is not uncommon to celebrate festivities in restaurants such as Samgyeopsal chains [75]. These consumers, despite non-frequent interactions with the brand or purchasing of products and services [76], generate high revenue [77]. Customers with higher income levels tend to have more purchasing ability as dictated in the study of [78].

#### 4.2.2 Core consumers

Core customers represent the cluster of consumers who eat Samgyeopsal at moderate frequency, meaning they do not classify as frequent consumers nor as less frequent consumers. They also represent consumers with a 15,000 Php—30,000 Php monthly allowance/salary. Similar to this group’s frequency of eating Samgyeopsal, the cluster’s salary is considered to be in the middle of the other two clusters’ salaries. Like the other two clusters, consumers in this cluster are considered to be in the age group of 15–24 years old. This cluster’s salary allows them to have enough financial comfort to consume Samgyeopsal in at a moderate, but not enough to consume the dish at a high frequency. The justification of this cluster’s age group is mostly because most participants in this research study are 15–24 years old. In addition, it can also be pointed towards the fact that most fans of Korean culture also fall within this age group [19]. This cluster allows businesses to have an ideal target market segment if they desire to appeal to most consumers.

#### 4.2.3 Low value consumers

Consumers with less than 15,000 PhP monthly allowance/salary are considered low valued customers. They are expected to have minor contributions among other segments due to their low capabilities to buy more compared to other consumers. Low-value customers also refer to those who buy the cheapest product of a company or restaurant or, relating to the study, the cheapest Samgyeopsal set the restaurant has to offer. In this regard, consumers in the financial bubble previously stated tend to buy less or buy cheaper. This concept relates to the buying behavior of consumers as directly affected by financial constraints. [79] discussed how choice restriction is an effect of financial constraints. The inevitability of being unable to afford options with higher prices was caused by this restriction [79]. Furthermore, the age bracket of 15–24 covers a wide array of occupations from students, part-timers, and fully employed workers. It is imperative that full-time students and part-time employees have limited finances, while those employed in this age had only begun working and are therefore also financially limited.

### 4.3 Marketing strategy

The importance scores dictated a large margin between the values of the upper-half and lower-half attributes. As a marketing strategy, Samgyeopsal should focus on promoting the attributes with higher importance values such as the choice of meat–heavily focusing on beef and pork, cheese inclusion, and price. Optimizing these attributes based on the segmentation provided in Fig 7 will result in higher rates of product consumption. Frequent consumers or high values customers include 15–24 years old with above 30,000 PhP monthly allowance/salary, while core customers have 15,000–30,000 monthly allowance/salary. Consistent contributions from these two classifications of customers greatly affect the profitability of Samgyeopsal restaurants, especially since it is a food business. Therefore, consistent tracking of service quality could suffice in enticing these high valued consumers to continuously buy the product.

In addition, consumers have a variety-seeking nature due to excessive familiarity with food. It would therefore be recommended to include different flavors of the meat to entice the consumers of Samgyeopsal. Proper modifications or additions to the restaurant’s offers, which include the use and variety of meat and cheese on the dishes are one effective method of improvement. For low-valued customers, promotions or discounts can attract consumers from this segment. It was previously mentioned in the findings that the younger generation is the likely consumer but has limitations with their budget. Promotions include student discounts or seasonal promotions that could reduce the burden of price on low values customers.

Lastly, Samgyeopsal is more commonly consumed by groups. Therefore, setting an environment suited for occasions and gatherings such as birthdays or celebrations can attract more customers. Moreover, in the pricing of Samgyeopsal offers, 499 PhP gained the highest utility estimate. This price level is the lowest among the options presented, thus is preferred by high values, core, and low values customers. The Samgyeopsal could use this to their advantage when promoting group promotions to attract more customers, with the same meat but of varied flavors.

### 4.4 Limitation and recommendation

With the high-value result of this study, there are still limitations present. First, this study was conducted during the COVID-19 pandemic. This may have caused for having less frequent consumption of Samgyeopsal per week due to the lockdown implemented. However, it was seen that there was still a significant number of Filipino customers who were willing to dine in Samgyeopsal restaurants. It is therefore recommended to conduct the study once the COVID-19 pandemic constraint has been lifted. Second, the consumers were mostly 15–24 years old. This is because the response for this study was collected online. [17] stated that even though an online survey is viable and sufficient for conjoint analysis, the result presented skewed responses among younger generations. Thus, diverse Samgyeopsal consumers may be considered to further justify the customer segmentation. Lastly, only preferences among Filipino consumers were considered in this study. A diverse result would be presented if future researchers collect and analyze data from different countries for a more general market segmentation of Samgyeopsal consumers.

## 5. Conclusions

The influence of Korean culture has started to be a global trend in recent years [1]. One of Korean culture’s famous dishes is the Samgyeopsal, a grilled dish where prepared ingredients are served, and customers construct the dish by individual preference [6]. This study utilized conjoint analysis to analyze combinations of attributes to determine the most considered attributes and K-means clustering to segment participants into different consumer clusters. There were 1,018 Samgyeopsal consumers who voluntarily participated in rating 27 attribute combinations of the Samgyeopsal dish. These considered attributes include the main meat, the addition of cheese, the cooking style, the price, the brand, and the drinks.

The conjoint analysis revealed that meat is the most important attribute in Samgyeopsal, wherein beef was the most preferred followed by pork. This was followed by preference with the addition of cheese, then price, drinks, cooking style, and then brands showed the least important attribute for Samgyeopsal. From the market segmentation, high-value customers were determined to have a 30,000 PhP and above monthly allowance/ salary and consume Samgyeopsal at high frequency. The core customers were ascertained to have a monthly allowance/salary of 15,000–30,000 PhP and consume Samgyeopsal with moderate frequency. Lastly, the low value customers were determined to have less than 15,000 PhP in monthly allowance/ salary and consume Samgyeopsal with moderate frequency. With that, the Samgyeopsal chains are recommended to create promotions in order to cater to different segments of consumers and gain more profit. This study is the first study that analyzed the preference of Filipinos for Samgyeopsal attributes. Finally, conjoint anlysis with k-means clustering can be utilized and extended for evaluating the food preference worldwide.
